# S-adenosylhomocysteine hydrolase-like protein 1 deficiency resulted in stronger inflammation reaction in acute pancreatitis

**DOI:** 10.1007/s00535-026-02437-x

**Published:** 2026-05-09

**Authors:** Juan Xiao, Wanlian Li, Jian Pan, Yinhui Yang, Ji-ao Chen, Xi-Dai Long

**Affiliations:** 1 Guangxi Key Laboratory of Molecular Medicine in Liver Injury and Repair, the Affiliated Hospital of Guilin Medical University, Guilin, 541001 Guangxi China; 2 Guangxi Health Commission Key Laboratory of Basic Research in Sphingolipid Metabolism Related Diseases, the Affiliated Hospital of Guilin Medical University, Guilin, 541001 Guangxi China; 3 Clinicopathological Diagnosis and Research Center, the Affiliated Hospital of Youjiang Medical University for Nationalities, Baise, 533000 China; 4 Departmemt of Basic Research, Key Laboratory of Tumor Molecular Pathology of Guangxi Higher Education Institutes, Baise, 533000 China

**Keywords:** Acute pancreatitis, AHCYL1, mTOR, Polyamine, Inflammation

## Abstract

**Background:**

The mechanism underlying acute pancreatitis (AP) is not fully understood. And the effect of s-adenosylhomocysteine hydrolase-like protein 1 (AHCYL1) in AP is unknown.

**Methods:**

In this research, AHCYL1 pancreatic acinar cell specific knockout (CKO) mice were constructed and AP was induced. The related mechanism was investigated in mice or primary cells.

**Results:**

It was found that AHCYL1 was decreased in AP mice. CKO caused increased macrophage infiltration in AP pancreas. AHCYL1 binding to amino acid transporters decreased in AP. Amino acid uptake, mTOR inhibition, and MIF production were aggravated in AP with AHCYL1 deficiency. Differentially expressed PGK1 in proteomics was associated with both mTOR and MIF in the PPI network. PGK1 was found to be further increased in CKO AP pancreas, which was reversed by mTOR activator. MIF expression and secretion under AP were inhibited by mTOR activator or PGK1 inhibitor. MIF-induced macrophage migration could be reduced by mTOR activator or PGK1 inhibitor. N1-acetylspermine increased in the serum of CKO AP mice. CKO acinar cell culture under AP caused further increment in N1-acetylspermine production enzyme, SSAT, which was inhibited by mTOR activator or PGK1 inhibitor. MIF induced SSAT and cytokines from macrophages, and the latter was reduced by SSAT inhibitor. Finally, AHCYL1 binding to the deubiquitinase OTUD6B decreased in AP. OTUD6B knockdown caused AHCYL1 ubiquitination and its decrement. AHCYL1 recovery reduced cytokine release and macrophage infiltration in AP.

**Conclusion:**

The AHCYL1 deficiency/amino acid uptake inhibition/mTOR inhibition/PGK1 up-regulation/MIF secretion axis in acinar cells induced SSAT in macrophages and macrophage infiltration in the pancreas in AP.

**Supplementary Information:**

The online version contains supplementary material available at https://doi.org/10.1007/s00535-026-02437-x.

## Background

Acute pancreatitis (AP) is a common inflammatory disease which occurs in the pancreas. Although AP is self-limited, severe cases will die since the mechanism underlying AP is not fully understood. For now, two main processes are involved in AP. Intracellular digestive enzymes pre-activation causes pancreatic acinar cell injury or cell death, leading to localized inflammation [1, 2]. Intercellular cytokines cause inflammatory cell infiltration into the pancreas and lead to systemic inflammation further [3]. Of note, digestive enzymes activation and inflammation could occur independently [1]. In the early stages of AP, macrophages play important roles in the inflammation [4].

AHCYL1 is known as IRBIT (IP_3_R binding protein released with inositol 1,4,5-trisphosphate), or DCAL. AHCYL1 protein localizes in the cytoplasm or the endoplasmic reticulum (ER) [5]. AHCYL1 can work with other proteins. It is reported that AHCYL1 binds to the IP_3_ (inositol 1,4,5-trisphosphate) receptor (IP_3_R) on the ER membrane to regulate calcium release from the ER [6, 7]. Besides, AHCYL1 is found to modulate ribonucleotide reductase to affect cell cycle progression [8]. Apart from proteins, AHCYL1 can bind to the metabolite, s-adenosylhomocysteine, and inhibit autophagy by decreasing the activity of phosphatidylinositol 3-kinase catalytic subunit Type 3 [9]. Besides, AHCYL1 is also found to regulate ion channels. It is reported that AHCYL1 interacts with sodium bicarbonate co-transporters NBCn1 at the cell plasma membrane, mimicking the effect of EGF [10]. Especially, AHCYL1 activates both the basolateral pNBC1 (electrogenic cotransporter) and the luminal CFTR to coordinate fluid and HCO_3_^−^ secretion by the pancreatic duct [11]. HCO_3_^−^ secretion deregulation is associated with pancreatitis [11]. However, the role of AHCYL1 in AP is not clear. Meanwhile, regulation of AHCYL1 on amino acid transportation is unknown.

Plasma amino acids alter in AP mice [12]. Besides, serum amino acid spectrum is disturbed in AP patients, which may be important for inflammation and organ function both in the pancreas and the gut [13]. Of note, amino acid transporters’ expression in acinar cells is changed during AP [14]. These reports indicate that intra acinar cells’ amino acids may be affected in AP. However, the direct relationship between amino acids and AP is not fully understood.

mTOR which is a serine/threonine protein kinase, plays a central role in cellular metabolism, growth and survival under hormones, growth factors, nutrients stress [15]. mTOR is inhibited when amino acids are insufficient [16]. In a recent research, protein phosphatase 2A inhibitors is reported to ameliorate AP by inhibiting the AKT-mTOR pathway to modulate macrophages [17]. It seems that mTOR is a stimulus for AP. However, it is found that mTOR is inhibited in the early stage of AP [18]. Therefore, the roles of mTOR in AP are not fully understood.

Polyamines including spermidine, spermine and N^1^-acetylspermine can be generated from amino acid metabolism [19]. These three metabolites can be converted into each other [19]. Polyamine catabolism is activated during the AP and the spermidine/spermine N^1^-acetyltransferase (SSAT) increment caused more severe AP [20, 21]. However, more details of polyamine metabolism in AP need to be illustrated.

In this research, the effects of AHCYL1 deficiency in pancreatic acinar cells in AP were investigated. The regulation network of amino acid, mTOR and polyamine metabolism in the case of AHCYL1 deficiency was also detected here.

## Materials and methods

### Chemicals and antibodies

The antibodies are used as follows: AHCYL1 (Proteintech), ATF4 (Proteintech), P-EIF2a (Proteintech), EIF2a (Proteintech), SLC7A5 (Proteintech), SLC7A8 (Affinity Biosciences), mTOR (CST), p-mTOR (CST), MIF (Abclonal), PGK1 (HUABIO), HMGB1 (HUABIO), LDHa (HUABIO), P65 (Abclonal), p-P65 (CST), SSAT (CST), PAOX (PTG), Amylase (Proteintech), Actin (Sangon Biotech), Tubulin (Proteintech), CD68 (Sangon Biotech), CD3(Proteintech), MPO(Sangon Biotech), Goat anti-rabbit IgG H&L (Alexa Fluor® 488) (abcam), Goat anti-mouse IgG H&L (Alexa Fluor® 647) (abcam).

The compounds are as follows: cerulein (MCE), lipopolysaccharide (LPS) (Solarbio), 3BDO (MCE), linoleic acid (MCE), docosatetraenoic acid (MCE), γ-linolenic acid (MCE), adenosine (MCE), palmitoylcarnitine (MCE), glycine (MCE), glutamic acid (MCE), tyrosine (MCE), kynurenic acid (MCE), diminazene aceturate (MCE), NG 52 (MCE), MIF (PTG), FITC-Tyrosine (Top-peptide Biotech), FITC-Leucine (Top-peptide Biotech), recombinant AHCYL1 (Origene).

### Animal experiments

AHCYL1 pancreatic acinar-cell-specific knockout mice were constructed by Cyagen Biosciences Inc in China. Briefly, the Cre-loxP system was applied. The sequence of the human PRSS1 promoter was fused with the Cre-encoding gene.

AP construction: Control or matched AHCYL1 CKO mice were induced by cerulein plus LPS or taurocholic acid (TA) according to our previous research [22]. Mice were sacrificed 3 h after the final injection of cerulein or 24 h after TA injection. Serum and pancreas were collected.

Compound treatment: AP was induced in control and AHCYL1 CKO mice. The solvent or the SSAT inhibitor diminazene aceturate (5 mg/Kg) was intraperitoneally injected into CKO mice with the first injection of cerulein. Mice were sacrificed 3 h after the final injection. Serum and pancreas were collected.

AHCYL1 recovery in mice: Method one, control or CKO mice (20 g, male) were treated with intraperitoneal injections of AAV2-vector or AAV2-AHCYL1 virus (1 × 10^13^ vg/mL) for 4 weeks. Then AP was induced using cerulein plus LPS or TA. Serum and tissue samples were collected as above. Method two, AHCYLl recombinant protein 1 mg/Kg was injected after first injection of cerulein, 0.1 mg/Kg AHCYL1 protein was injected after the TA injection.

### HE staining

Pancreas tissues were fixed with paraformaldehyde, then dehydrated and embedded into paraffin. Samples were processed, and 4-µm thick paraffin sections were stained with hematoxylin and eosin (H&E).

### Immunochemistry

Pancreas tissues were fixed with paraformaldehyde and embedded in paraffin. Tissue sections on the histological slides were dewaxed, dehydrated, antigen-repaired, incubated with peroxidase blocking reagent, blocked with serum blocking reagent, incubated with primary antibodies followed by secondary antibodies, then incubated in DAB staining solution, finally counterstained with the nuclear counterstain hematoxylin.

### Biochemical assay

Serum samples were diluted and then were detected with an automatic biochemical analyzer (Mindray).

### Cytokine measurement

Serum or cell culture samples were diluted and then measured using a BCA cytokine kit (BD) by a flow cytometer (Thermo Fisher Scientific).

### ELISA

MIF in serum or cell culture was detected by an ELISA kit (PTG) according to the manufacturer instruction.

### Primary cell purification

For pancreatic acinar cells, the pancreas was cut into small pieces and digested with collagenase at 37 °C with 5% CO_2_. Acinar cells were obtained after digestive mixture filtering. Acinar cells were cultured in DMEM medium with 15% FBS at 37 °C and 5% CO_2_.

For macrophages: mice were intraperitoneally injected with 4 mL of 3% thioglycollate medium for 96 h. Mice were sacrificed and intraperitoneal macrophages were resuspended in cold PBS. PBS with macrophages was collected and centrifuged. Macrophages were obtained and cultured in DMEM medium with 10% FBS at 37 °C and 5% CO_2_.

### Metabolomics and proteomics assay

Cell cultures, serum or pancreas tissues under the indicated treatments were sent to Shanghai Baipu Biotechnology Co. for metabolomics or proteomics.

### Amino acid uptake

Pancreatic acinar cells were starved for 30 min and then were treated with 1 mM FITC-Tyrosine or FITC-Leucine for 20 min. Cells were washed and sent for imaging with a fluorescence microscope or a flow cytometer to detect intracellular amino acid content.

### Co-immunoprecipitation

Pancreas from saline or cerulein-plus-LPS-treated mice were lysed with IP lysis buffer (Beyotime Biotechnology) containing protease and phosphatase Inhibitor Cocktail (MCE). Lysates were centrifuged and the supernatant was incubated with anti-AHCYL1 antibody at 4 °C overnight. Protein A/G Magnetic Beads (Thermo Scientific Pierce) were added. Finally, beads were enriched and washed. Proteins binding to AHCYL1 on the beads were analyzed by mass spectrometry and western blotting.

### Immunofluorescence

Cells were fixed with 4% paraformaldehyde and then were penetrated with 0.1% triton-X 100. Pancreas tissues were fixed with 4% paraformaldehyde and embedded into paraffin. Tissue sections on the histological slides were dewaxed, dehydrated, antigen-repaired, blocked with a serum blocking reagent. Cells and tissue were blocked with 5% goat serum. AHCYL1 antibody was added to cells at 4 °C overnight. Secondary antibody to rabbit IgG H&L (Alexa Fluor® 488) was added to cells at room temperature for 1 h. Then SLC7A5, SLC7A8 or OTUD6B antibody and secondary antibody to mouse IgG-H&L (Alexa Fluor® 647) were added as for AHCYL1. SLC7A5, SLC7A8 or OTUD6B and AHCYL1 co-localization was imaged with fluorescence microscopy.

### PPI network construction

Differentially expressed proteins in the pancreas from AHCYL1 CKO mice with AP compared with the control pancreas together with mTOR were put into the online analyzer (https://string-db.org/). Then the PPI network was downloaded.

### Transwell assay

Pancreatic acinar cells under the indicated treatment were co-cultured with macrophages in the upper chamber. The lower compartment contained DMEM with 15% FBS and the upper compartment contained only DMEM. After 24 h, cells in the upper cells were fixed with paraformaldehyde and stained with 0.1% crystal violet. Non-migrating cells were removed before imaging. The number of migrated cells in each well was calculated using ImageJ as the mean value from the results of six fields.

### Western blotting

Cell or tissue lysates were separated in SDS-PAGE. Proteins in the gel were transferred to a PVDF membrane. After being probed with primary antibodies followed by HRP-conjugated secondary antibodies. Then the signaling of target proteins were visualized by ECL substrate.

### Statistical analysis

The relationship between the expression content of MIF and HMGB1, PGK1 or LDHa was analyzed by linear regression. Graphs in each figure were represented as mean ± standard deviation (SD). Significant differences between two groups were evaluated using an independent Student’s t test in SPSS software. Significant differences among three or more groups were evaluated using one-way ANOVA in SPSS software. The pairwise comparisons were conducted using LSD or Tamhane analysis. ^*^p < 0.05, ^**^p < 0.01, ^***^p < 0.001.

## Results

### AHCYL1 decreased in acute pancreatitis

**Fig. 1:**
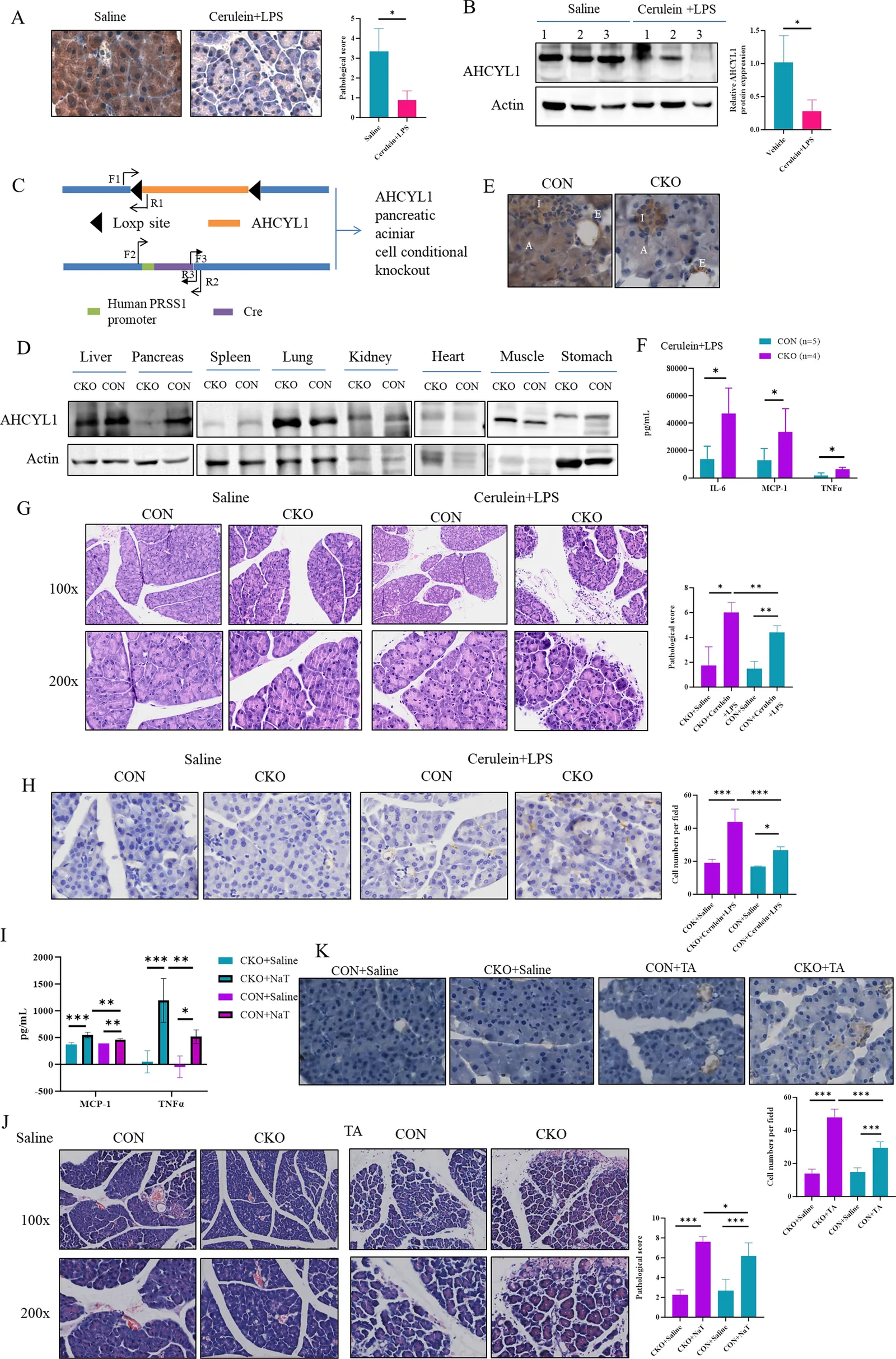
Pancreatic deficiency of AHCYL1 resulted in severer inflammation during AP.** A**, **B** AP was induced in C57BL/6 mice (*n* = 3 in each group), 3 h after the final injection of cerulein, mice were sacrificed and pancreas tissues were collected. Part of the pancreas was fixed and the tissue sections were sent for immunochemistry with AHCYL1 antibody, the representative images were shown (**A**). The other part of the tissue was lysed and sent for western blotting with AHCYL1 and Actin antibodies, the blot intensity was quantified using ImageJ (**B**). (**C**–**K**) AHCYL1 conditional knockout mice were constructed. The summarized protocol was shown (**C**). Indicated tissues collected from control or AHCYL1 CKO mice were analyzed by western blotting (**D**) or immunochemistry (**E**). AP was induced in control or AHCYL1 CKO mice (**F**–**K**). Serum was collected and cytokines were detected with flow cytometer (**F** and **I**). Pancreas tissues were embedded and tissue sections were sent for HE staining (**G** and **J**) and immunochemistry (**H** and **K**). CD68-positive macrophage number in each sample was evaluated as mean value from five fields using ImageJ (H and K). *A* acinar cells, *I* islet, *E* endothelial cells. Graph is presented as mean ± SD, ^*^p < 0.05, ^**^p < 0.01, ^***^p < 0.001

To investigate the potential relationship between AHCYL1 and AP, AP in mice was constructed by injection with cerulein plus LPS. AHCYL1 protein expression in pancreatic tissues from AP mice was tested. IHC staining results showed that AHCYL1 was present in the cytoplasm of acinar cells (Fig. 1A). Compared to the saline treatment, cerulein plus LPS reduced the content of AHCYL1 (Fig. 1A). Western blotting was also performed and indicated that AHCYL1 protein decreased in the pancreas with AP (Fig. 1B).

To investigate the role of AHCYL1 in AP further, AHCYL1 conditional knockout mice were established using the Cre-Loxp system. According to the previous report, the human PRSS1 promoter could successfully induce many genes’ expression in mice [23]. Meanwhile, PRSS1 is specially expressed in pancreatic acinar cells. Therefore, this promoter was applied for AHCYL1 pancreas-specific knockout (AHCYL1 CKO) mice construction (Fig. 1C). The genotype of the mice (Figure S1) and the protein level of AHCYL1 expression were investigated (Fig. 1D). AHCYL1 protein was found to decrease especially in the pancreas (Fig. 1D). Specifically, AHCYL1 was knocked out in pancreatic acinar cells (Fig. 1E).

AP was induced with cerulein plus LPS or TA in control and AHCYL1 CKO mice. Serum amylase and lipase, which were the clinical diagnosis predictors for AP [24], were detected first. It was found that these two markers were similar in control and AHCYL1 CKO mice under saline or cerulein plus LPS treatment (Figure S2A and S2B). Inflammatory reaction is another important character of AP. The results showed that pro-inflammatory cytokines including IL-6, MCP-1 and TNFα did not show a significant difference between control and AHCYL1 CKO mice under saline treatment (Figure S2C). However, these cytokines were increased in AHCYL1 CKO mice in both cerulein plus LPS and TA-induced AP (Fig. 1F and 1I). Meanwhile, compared to the control, more inflammatory cell infiltration resulting in more injury in the pancreas from AHCYL1 CKO mice with AP was observed (Fig. 1G and 1J). Many types of inflammatory cells including macrophages were involved in the inflammatory reaction during the AP [25]. Therefore, we investigated the presence of macrophages in AHCYL1 CKO mice with AP. The IHC results showed that CD68 + macrophages infiltrating the pancreas increased in AP, and the cells increased further in AHCYL1 CKO mice (Fig. 1H and 1K). Of note, AHCYL1 did not affect T cells or neutrophils in the pancreas (Figure S3). These results indicated that AHCYL1 knockout resulted in a stronger inflammatory reaction in AP.

### AHCYL1 knockout caused inhibition further in acinar cells’ amino acid uptake

**Fig. 2:**
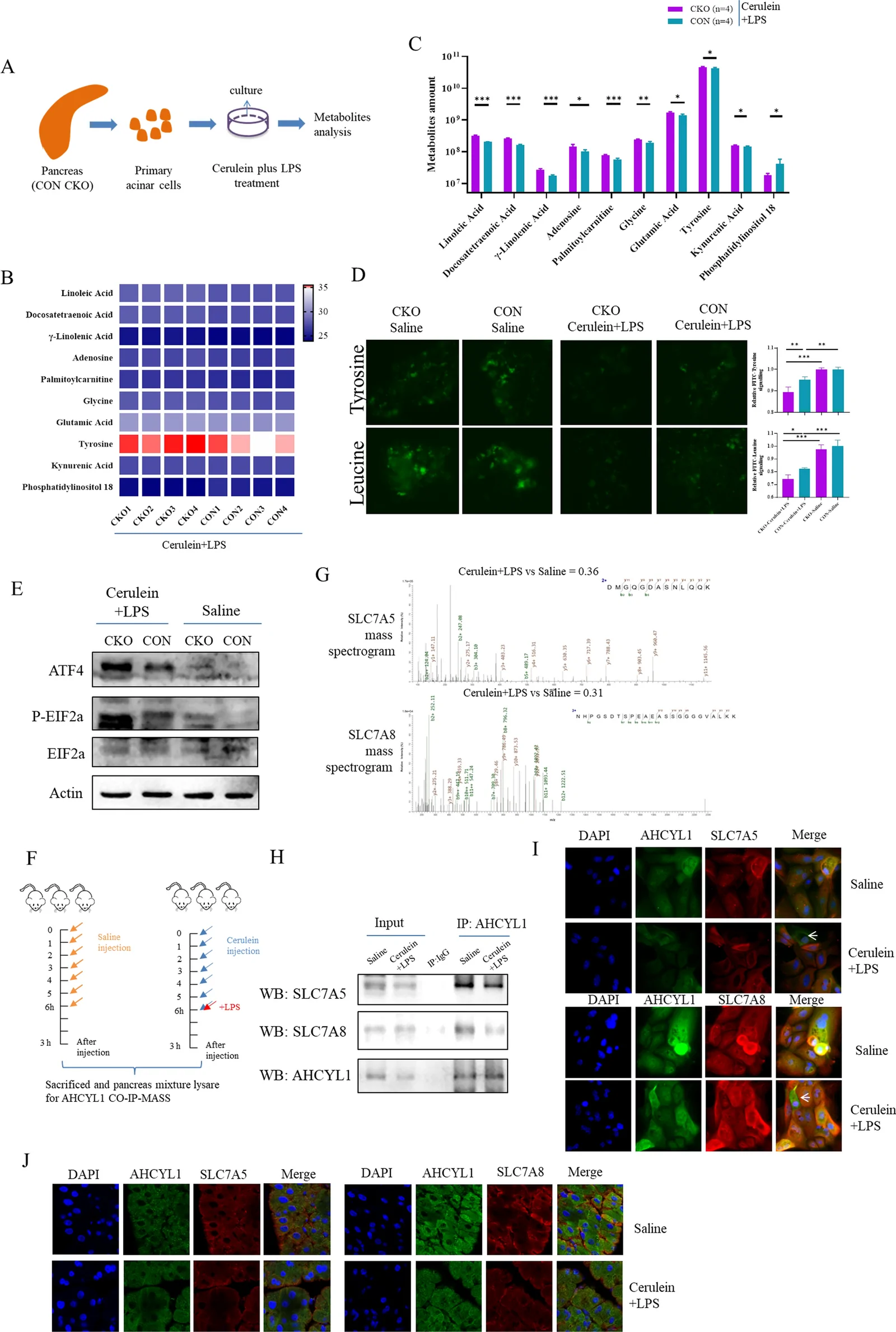
Pancreatic deficiency of AHCYL1 resulted in more severe amino acids uptake inhibition during AP. **A**–**C** Cerulein plus LPS-treated acinar cell culture was collected and sent for metabolomics analysis. The workflow was listed (**A**). The signal value of the amount of indicated metabolites was transformed to log2 and visualized as a heat map (**B**). Metabolite content was shown as a histogram (**C**). **D** Acinar cells were sent for amino acids uptake assay, intracellular amino acids signaling was analyzed and the mean intensity value ± SD was shown in the graph. ^*^p < 0.05, ^**^p < 0.01, ^***^p < 0.001. **E**, **I** Primary pancreatic cells were isolated and cells were treated with saline or 200 nM cerulein plus 10 ng/mL for 6 h. Cells were fixed and sent for western blotting (**E**) and immunofluorescence assay (**I**). **F**–**H**, **J** AP was induced in C57BL/6 mice. Pancreas tissues from the saline or cerulein plus LPS group were lysed, and lysates were incubated with AHCYL1 antibodies. AHCYL1 binding proteins were enriched and sent for mass spectrum analysis (**F** and **G**), western blotting (H), or immunofluorescence (**J**). The signaling ratios of target proteins binding to AHCYL1 under cerulein plus LPS versus saline were calculated and listed with the mass spectrogram (**G**)

The above results suggested that AHCYL1 deficiency aggravated AP. Of note, AHCYL1 was selectively knocked out in the pancreas (pancreatic acinar cells). Therefore, components secreted from the pancreas might probably contribute to the increase in macrophage infiltration in AHCYL1 CKO mice with AP. In order to figure it out, primary pancreatic acinar cell culture was analyzed by metabolomics. The workflow was shown here (Fig. 2A). The results showed that metabolites including linoleic acid, docosatetraenoic acid, γ-linolenic acid, adenosine, palmitoylcarnitine, glycine, glutamic acid, tyrosine, kynurenic acid, phosphatidylinositol 18 changed significantly in AHCYL1 CKO mice. The pattern of the metabolites secreted from control and AHCYL1 CKO pancreatic acinar cells under cerulein plus LPS treatment was shown in the heat map (Fig. 2B). Apart from phosphatidylinositol 18, the other metabolites increased in cell culture from AHCYL1 CKO acinar cells (Fig. 2C). It should be mentioned here that mice injected with the increased metabolites under AP did not show an increase in cytokine release. Therefore differential metabolites might not be the reason for the inflammatory reaction induced by AHCYL1 deficiency.

Of note, among these metabolites, three amino acids including glycine, glutamic acid and tyrosine were present (Fig. 2B and 2C). It was hypothesized that AHCYL1 might be involved in amino acid uptake during AP. In order to validate this hypothesis, we performed an amino acid uptake experiment with green fluorescence conjugated tyrosine, which is a non-essential amino acid. Tyrosine decreased in control acinar cells under cerulein plus LPS treatment and it decreased further in AHCYL1 CKO acinar cells (Fig. 2D). Besides, the uptake of leucine, which is an essential amino acid and was absent in the metabolomics data, was also detected. Consistently, leucine uptake was inhibited under AP conditions and AHCYL1 deficiency made this situation worse (Fig. 2D). Moreover, protein synthesis-related proteins, including ATF4 and P-EIF2α, were increased further in cerulein plus LPS-treated AHCYL1-deficient acinar cells (Fig. 2E). Similar changes were found in amylase (Figure S4). Taken together, AHCYL1 was closely related to amino acid uptake in AP.

### AHCYL1 binds to amino acid transporters SLC7A5 and SLC7A8

The mechanism underlying AHCYL1 deficiency-induced amino acid uptake inhibition needs to be investigated. Pancreas tissue from mice with saline or cerulein plus LPS treatment was lysed and AHCYL1 in the pancreas lysate was purified with the indicated antibody. The co-immunoprecipitation components were analyzed by mass spectrum (Fig. 2F). Compared to saline, the amounts of amino acid transporters including SLC7A5 and SLC7A8 were reduced in binding to AHCYL1 under cerulein plus LPS treatment (Fig. 2G). The signaling ratios (cerulein + LPS vs saline) of SLC7A5 and SLC7A8 binding to AHCYL1 were 0.36 and 0.31 respectively (Fig. 2G).

To validate the results from mass spectrometry, Western blotting was performed. Interactions between SLC7A5 or SLC7A8 and AHCYL1 under saline treatment were found (Fig. 2H). However, cerulein plus LPS could reduce their interactions (Fig. 2H). Moreover, we visualized the localization of SLC7A5, SLC7A8 and AHCYL1 in pancreatic acinar cells and the pancreas. The co-localization of AHCYL1 and SLC7A5 could be observed in acinar cells and the pancreas treated with saline (Fig. 2I and 2J). However, more non-binding protein signaling (white arrow) in cells (Fig. 2J) or less orange signaling in the pancreas (Fig. 2J) with cerulein plus LPS were found. A similar phenomenon was found in the co-localization of AHCYL1 and SLC7A8 in cells and the pancreas (Fig. 2I and 2J). These results suggested that decreased AHCYL1 binding to SLC7A5 or SLC7A8 was related to AP occurrence. It is known that SLC7A5 and SLC7A8 are responsible for amino acid transport, including glycine, tyrosine, and leucine [26, 27]. Taken together, AHCYL1 knockout induced the downregulation of the AHCYL1-SLC7A5/SLC7A8 complex, which might contribute to a further decrement in amino acid uptake in AP.

### AHCYL1 deficiency inhibited mTOR activation

**Fig. 3:**
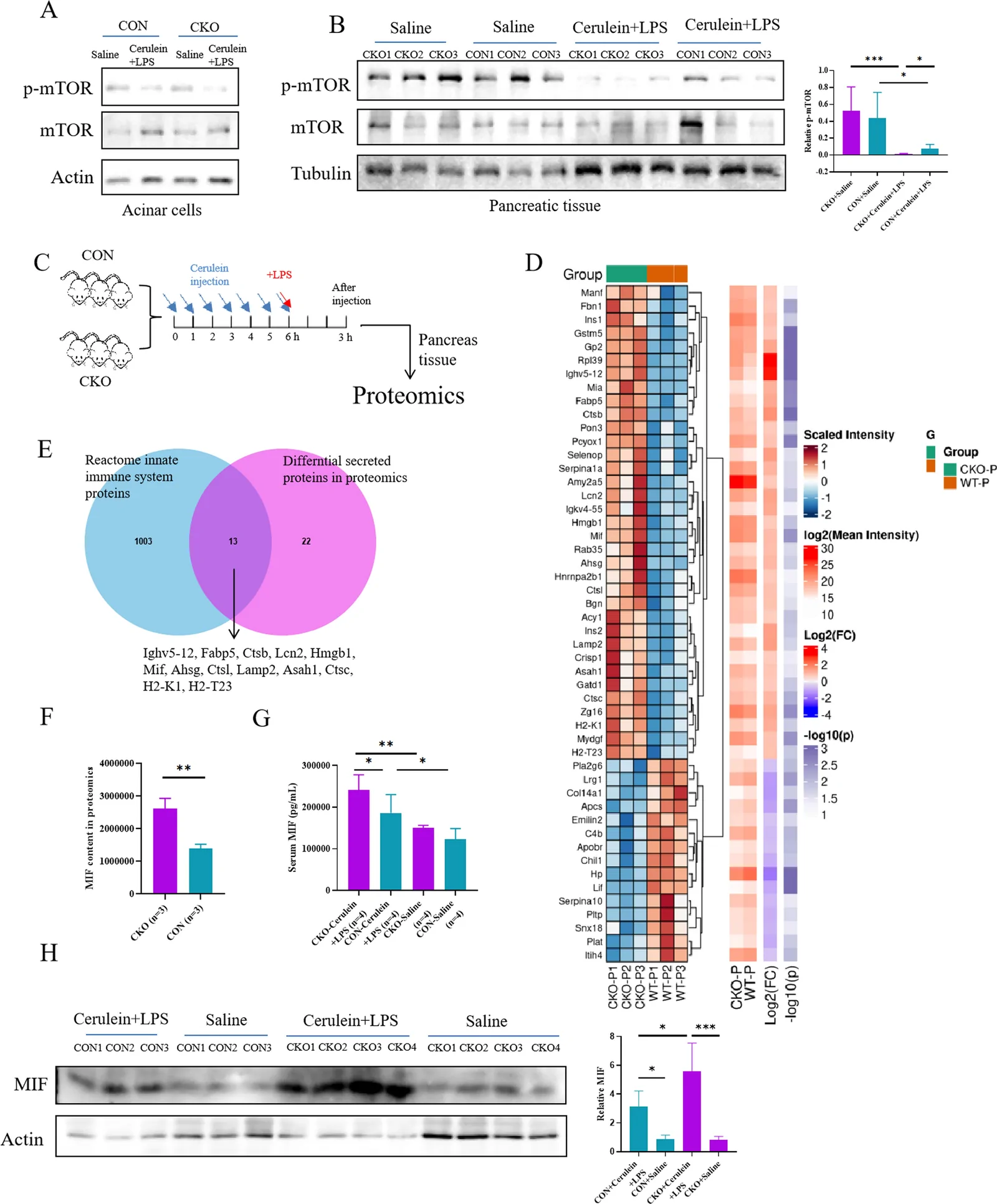
AHCYL1 deficiency caused stronger mTOR inhibition and MIF expression in the pancreas during AP. **A** Primary pancreatic cells were treated with saline or 200 nM cerulein plus 10 ng/mL for 6 h. Cells were lysed and sent for western blotting with the indicated antibodies. **B**–**H** AP was induced in control and AHCYL1 CKO mice. Pancreas tissues were lysed and lysates were sent for western blotting with the indicated antibodies. Protein intensity was quantified by ImageJ (**B** and **H**). **C**–**F** Pancreases from cerulein plus LPS mice were sent for proteomics analysis. Differentially expressed secretory protein content is shown as the heat map (**D**). A Venn diagram of innate immune system-related proteins and the 35 top differential secreted proteins was done on https://jvenn.toulouse.inrae.fr/app/example.html (**E**). MIF amount was shown in the histogram (**F**). Serum was collected and sent for ELISA assay (**G**). Graphs are presented as mean ± SD, ^*^p < 0.05, ^**^p < 0.01, ^***^p < 0.001

AHCYL1 deficiency led to inhibition of uptake of both nonessential and essential amino acids. Meanwhile, amino acid deprivation inhibits mTOR activation [16]. Therefore, mTOR might be involved in AP induced in AHCYL1 CKO mice. Primary pancreatic acinar cells were investigated first. Acinar cells isolated from control or AHCYL1 CKO mice were treated with saline or cerulein plus LPS. The results showed that cerulein plus LPS decreased the level of mTOR phosphorylation in both types of acinar cells (Fig. 3A). Compared to the control, AHCYL1 CKO acinar cells showed lower levels of phosphorylated mTOR under cerulein plus LPS treatment (Fig. 3A). Moreover, mTOR phosphorylation was investigated in the pancreas. Consistently, mTOR phosphorylation decreased in the pancreas from AP mice and it showed a further decrement in the pancreas of AHCYL1 CKO mice with AP (Fig. 3B). These results suggested that AHCYL1 deficiency caused further inhibition of mTOR activation.

### AHCYL1 deficiency promoted MIF production

Macrophage infiltration in the pancreas increased further in AHCYL1 CKO mice with AP. Metabolites secreted from the pancreas in AHCYL1 CKO mice might not be the direct stimulus for this phenomenon. In order to elucidate this problem, pancreas from control or AHCYL1 CKO mice with AP were sent for proteomic analysis (Fig. 3C). Secreted proteins with significant changes in proteomics data were selected and their amounts in each sample were visualized as a heat map (Fig. 3D). Among the up-regulated proteins (the top 35), we found 13 proteins, including Ighv5—12, Fabp5, Ctsb, Lcn2, Hmgb1, Mif, Ahsg, Ctsl, Lamp2, Asah1, Ctsc, H2-K1, H2-T23, present in the innate immune-related proteins from the Reactome database (Fig. 3E). We focused on proteins in acinar cells and Fabp5, Ctsb, Lcn2, Hmgb1 and MIF were investigated next. MIF was found to be the cytokine closely related to macrophage recruitment [28]. Its amount increased by nearly one fold in the AHCYL1 CKO pancreas compared to the control in the proteomics analysis (Fig. 3F). Then MIF was detected in vivo. Serum MIF increased in AP mice, and more MIF was found in AHCYL1 CKO mice with AP compared to the control (Fig. 3G). Pancreatic MIF showed similar changes with serum MIF (Fig. 3H). However, the other proteins did not change significantly between the control and AHCYL1 deficiency. The results indicated that AHCYL1 deficiency promoted MIF production. MIF might contribute to macrophage infiltration in AHCYL1 CKO mice.

### AHCYL1 deficiency strengthened mTOR/PGK1 axis in AP

**Fig. 4:**
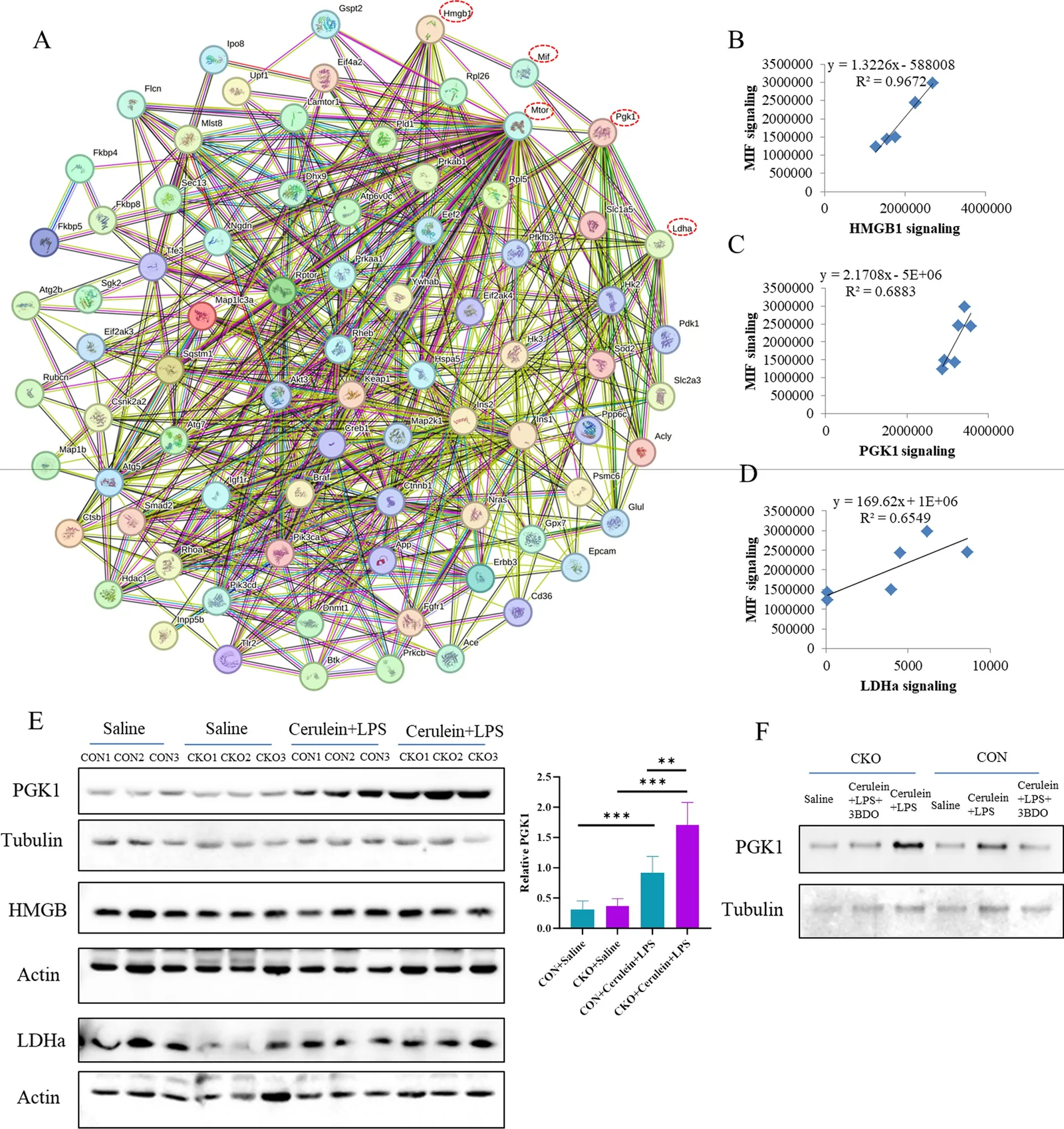
AHCYL1 deficiency resulted in stronger mTOR/PGK1 signaling transduction during AP. **A** PPI network among mTOR and differentially expressed proteins in proteomics data was conducted. **B**–**D** The amount values of MIF and HMGB1, PGK1 or LDHa in proteomics data were sent for linear regression. **E** AP was induced in control and AHCYL1 CKO mice, pancreas tissues were lysed and lysate were sent for western blotting with indicated antibodies, protein intensity qualified by ImageJ shown in the graph presented as mean ± SD. Protein intensity, quantified by ImageJ, is shown in the graph presented as mean ± SD. ^**^p < 0.01, ^***^p < 0.001. **F** Primary pancreatic cells were treated with saline, 200 nM cerulein plus 10 ng/mL or cerulein plus LPS and 10 μM 3BDO for 6 h. Cells were lysed and sent for western blotting with indicated antibodies

AHCYL1 deficiency inhibited mTOR and promoted MIF. The relationship between mTOR and MIF was investigated next. Of note, AHCYL1 deficiency did not affect the protein level of mTOR in the proteomics data. Proteins significantly differentially presented in AHCYL1 CKO pancreas, compared to the control and mTOR, were analyzed on the protein–protein interaction (PPI) Networks website. The analysis results suggested that HMGB1, PGK1 and LDHa correlated to both MIF and mTOR (red circle) and there is no direct connection between mTOR and MIF (Fig. 4A). Moreover, linear regression was performed. HMGB1, PGK1, or LDHa with MIF showed good linear association according to the proteomics data (Fig. 4B–D). HMGB1, PGK1 and LDHa were tested in the pancreas. It was found that PGK1 protein increased in AP mice and it increased further in AHCYL1 CKO mice with AP (Fig. 4E). Meanwhile, HMGB1 and LDHa did not change significantly under the indicated treatments (Fig. 4E). Therefore, PGK1 might probably correlate to MIF. Moreover, the relationship between PGK1 and mTOR was investigated. In primary pancreatic acinar cells, PGK1 expression increased in cerulein-plus-LPS-treated cells and it increased further in AHCYL1 CKO acinar cells (Fig. 4F). mTOR activator 3BDO inhibited the effect of cerulein plus LPS on PGK1 (Fig. 4F). These results indicated that mTOR inhibition induced PGK1 in AP and AHCYL1 further strengthened this regulation.

### AHCYL1 deficiency strengthened mTOR/PGK1/MIF/macrophage migration axis in AP

**Fig. 5:**
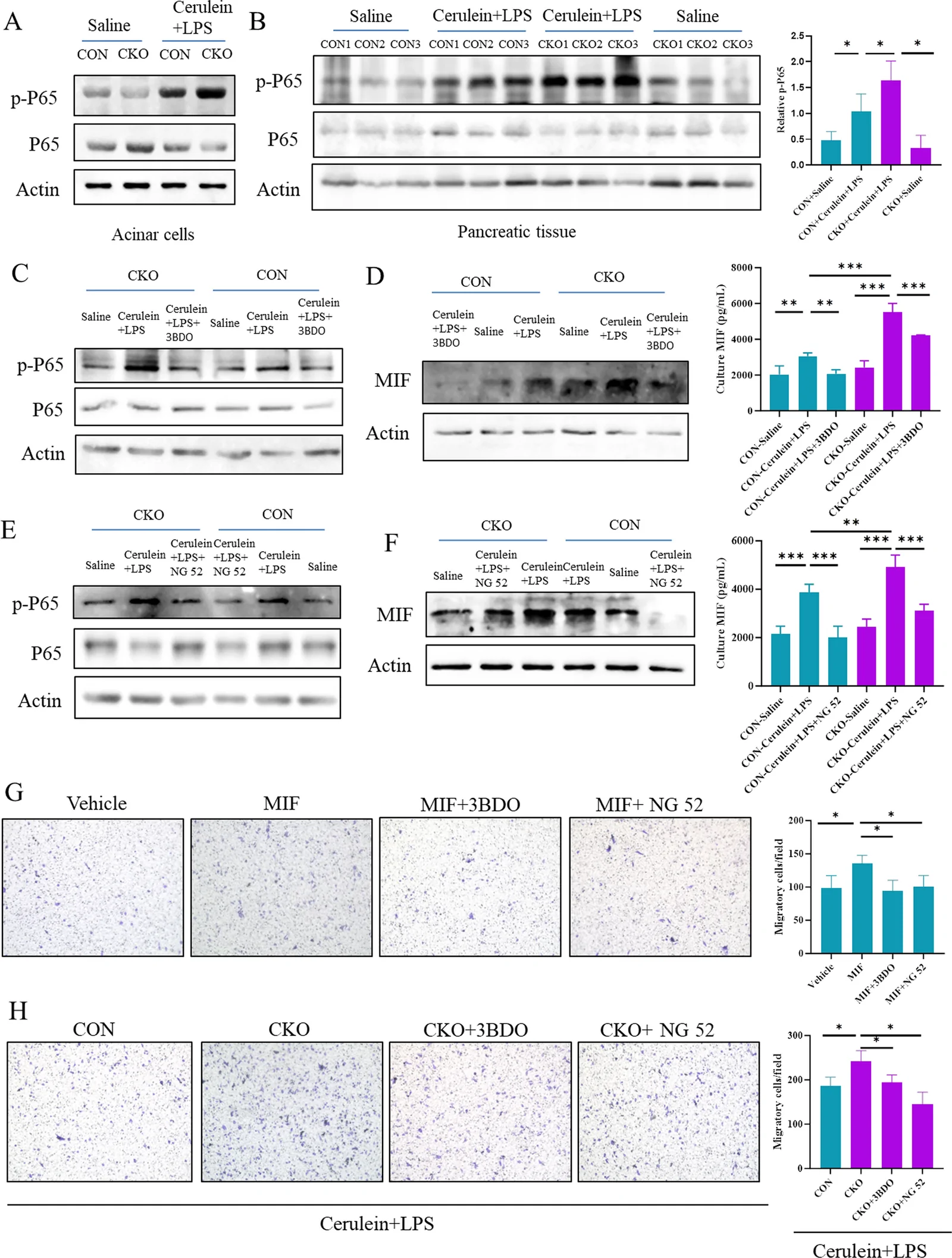
AHCYL1 deficiency resulted in stronger mTOR/MIF signaling transduction during AP. **A** Primary pancreatic cells were treated with saline or 200 nM cerulein plus 10 ng/mL for 6 h. Cells were lysed and sent for western blotting with indicated antibodies. **B** AP was induced in control and AHCYL1 CKO mice, pancreas tissues were lysed and lysate were sent for western blotting with indicated antibodies, protein intensity was qualified by ImageJ. **C**–**F** Primary pancreatic cells were treated with saline, 200 nM cerulein plus 10 ng/mL or cerulein plus LPS and 10 μM 3BDO (or NG 52) for 6 h. Cells were lysed and sent for western blotting with indicated antibodies (**C** and **D** left, **E** and **F** left). MIF was measured by ELISA (**D** right and **F** right). **G**, **H** Macrophages were seeded in the upper chamber, indicated reagents were added to lower chamber with acinar cells (**H**) or without acinar cells (**G**) for 24 h, upper chamber cells were fixed and migrated cells were calculated and mean cell numbers ± SD shown in the graph. Graph presented as mean ± SD, ^*^p < 0.05, ^**^p < 0.01, ^***^p < 0.001

The regulation of mTOR/PGK1 on MIF expression was investigated. It is known that NF-kb induces cytokine expression in AP [29, 30]. Therefore, NF-kb, whose activation was characterized as P65 phosphorylation (p-P65), was first tested. It was found that p-P65 increased in cerulein plus LPS treated acinar cells and p-P65 increased further in AHCYL1 CKO acinar cells (Fig. 5A). Meanwhile, p-P65 showed similar changes in pancreas (Fig. 5B). Moreover, mTOR activator 3BDO decreased p-P65 induced by cerulein plus LPS (Fig. 5C). MIF expression was tested next. MIF increased in acinar cells under cerulein plus LPS treatment and it increased further in AHCYL1 CKO acinar cells (Fig. 5D left). Of note, MIF expression in acinar cells was validated by single-cell sequencing (Figure S5). To our expectation, mTOR activator decreased MIF induced by cerulein plus LPS (Fig. 5D left). MIF in acinar cell culture showed consistent changes (Fig. 5D, right). Therefore, mTOR inactivation induced MIF production. PGK1 inhibitor was also applied. The levels of p-P65 and MIF induced by cerulein plus LPS in acinar cells were also reduced in the presence of PGK1 inhibitor NG 52 (Fig. 5E). MIF expression and secretion showed similar changes (Fig. 5F). These results indicated that PGK1 promoted MIF production. Taken together, mTOR inhibition induced PGK1 followed by MIF up-regulation. mTOR/PGK1 axis regulation on MIF might be through NF-kb.

The above results showed that AHCYL1 deficiency caused more macrophage infiltration in the pancreas in AP. Meanwhile, it is reported that MIF could induce macrophage migration. Therefore, the effect of the mTOR/PGK1 axis on macrophage migration was investigated. MIF was added to macrophages directly. Both the mTOR activator and the PGK1 inhibitor could reduce macrophage migration induced by MIF (Fig. 5G). Besides, an acinar cell–macrophage co-culture system was constructed. The results showed that cell culture from cerulein-plus-LPS-treated acinar cells induced macrophage migration and AHCYL1 deficiency made it stronger (Fig. 5H). Conversely, both the mTOR activator and the PGK1 inhibitor could inhibit macrophage migration (Fig. 5H). Therefore, the mTOR/PGK1 axis could regulate macrophage migration.

### AHCYL1 deficiency-induced SSAT through mTOR/PGK1/MIF in macrophages in AP

**Fig. 6:**
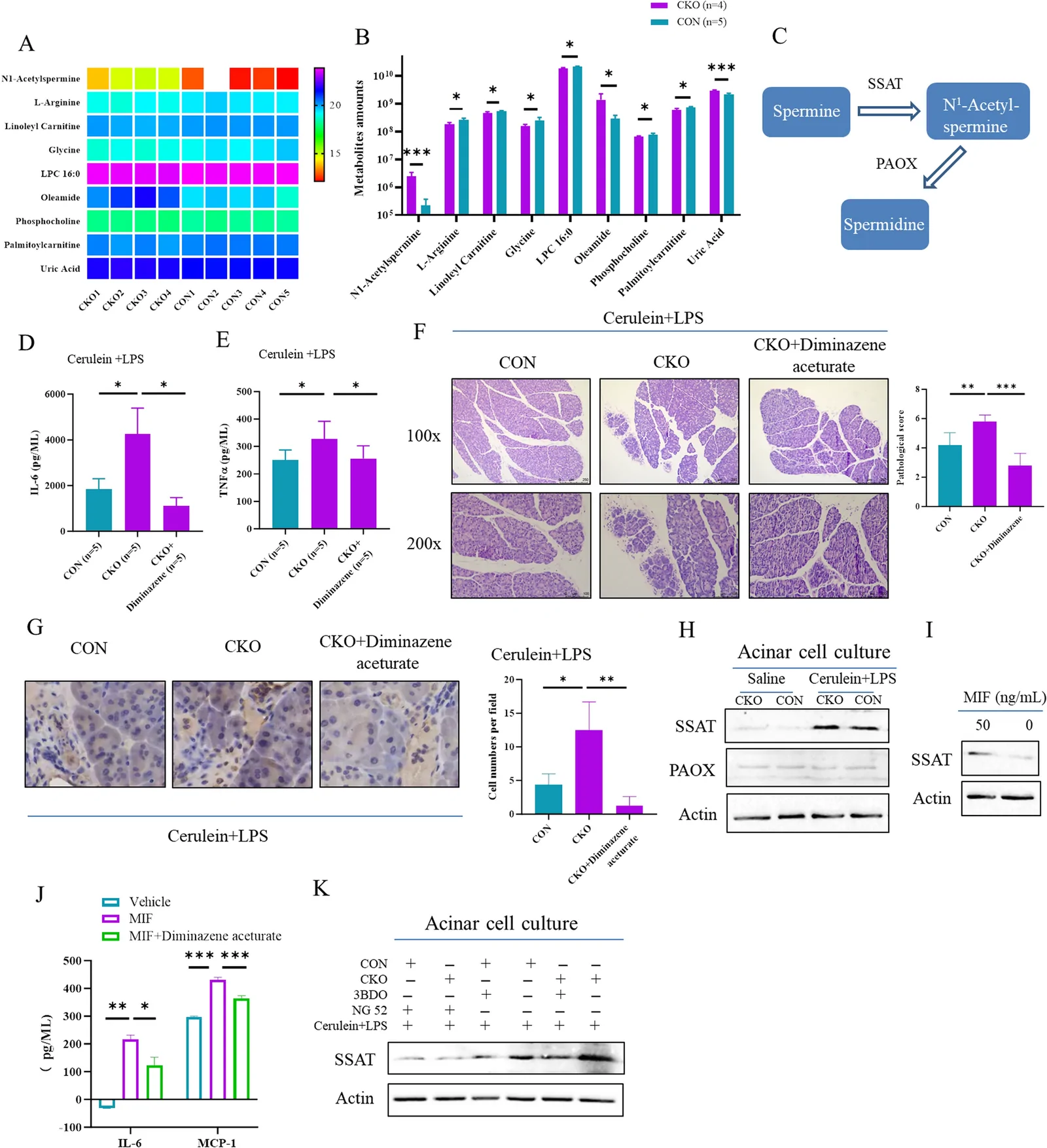
AHCYL1 deficiency resulted in stronger mTOR/PGK1/MIF/SSAT signaling transduction during AP. **A**, **B** AP was induced in control and AHCYL1 CKO mice, serum were collected and sent for metabolomics analysis. The amounts of differential metabolites were visualized as the heat map (**A**) and histogram (**B**). N^1^-Acetylspermine metabolic pathways were summarized (**C**). **D**–**G** Control mice were injected with cerulein plus LPS. AHCYL1 CKO mice were injected with cerulein plus LPS in the presence or absence of Diminazene aceturate. Cytokines in serum were detected with a flow cytometer (**D** and **E**). Pancreas tissue sections were sent for HE staining (**F**) and immunohistochemistry (**G**). The number of CD68-positive macrophages in each sample was evaluated as the mean value from five fields using ImageJ (**G**).** H**, **K** Primary pancreatic cells were treated as indicated. Acinar cell culture was collected and added to macrophages. Macrophages were lysed and lysates were sent for western blotting with the indicated antibodies. **I**, **J** Macrophages were treated with or without 50 ng/mL MIF in the presence or absence of Diminazene aceturate. Macrophages were lysed and lysates were sent for western blotting with the indicated antibodies (**I**). Cell culture was collected and cytokines were measured. Graphs are represented as mean ± SD, ^*^p < 0.05, ^**^p < 0.01, ^***^p < 0.001

We investigated the mechanism underlying AHCYL1 deficiency caused inflammation increment in AP further. Serum from control and AHCYL1CKO mice with AP was sent for metabolomics analysis. Differential metabolites were shown (Fig. 6A and 6B). Each metabolite in the indicated samples was displayed in the heat map (Fig. 6A). The mean value of the amount of each metabolite in the two groups was listed, and N^1^-acetylspermine was found to be the increased metabolite with the largest fold in AHCYL1 CKO mice (Fig. 6B).

According to the previous reports, N^1^-acetylspermine can be produced from spermine by spermidine/spermine N1-acetyltransferase (SSAT) [19]. Meanwhile, it can be hydrolyzed into spermidine by N1-acetylspermine (PAOX) [19]. The SSAT inhibitor diminazene aceturate [31] was used in AP. It was found that SSAT inhibition reduced cytokines in serum from AHCYL1 CKO mice with AP (Fig. 6D and 6E). Meanwhile, SSAT1 inhibitor decreased inflammatory cell infiltration in the pancreas in AHCYL1 CKO mice (Fig. 6F). Specifically, the amount of CD68 + macrophages in the pancreas was reduced in AHCYL1 CKO mice with AP in the presence of the SSAT inhibitor (Fig. 6G). These results suggested that increased macrophage infiltration in AHCYL1 CKO mice was due to SSAT.

Next we investigated how SSAT was regulated. The protein expression of SAAT and PAOX in acinar cells was first tested. Pancreatic acinar cells from control or AHCYL1 CKO mice were treated with saline or cerulein plus LPS. The two proteins did not change significantly under the indicated treatments. Of note, the AHCYL1 deficient acinar cell culture under cerulein plus LPS treatment induced SSAT1 without affecting PAOX in macrophages (Fig. 6H). The above results showed that nine metabolites were increased in AHCYL1 CKO acinar cell culture (Fig. 2C). These metabolites were added to macrophages directly. The metabolites did not significantly affect SAAT and PAOX in cells (Figure S6). Since differential metabolites did not affect SSAT in macrophages, other types of components like proteins might work. Therefore, MIF was directly treated with MIF. It was found that MIF could result in SSAT up-regulation in macrophages (Fig. 6I). Moreover, exogenous MIF caused cytokine release from macrophages and the SSAT inhibitor reversed this effect (Fig. 6J). Besides, culture from pancreatic acinar cells treated with cerulein plus LPS in the presence of an mTOR activator or a PGK1 inhibitor reduced SSAT protein expression in macrophages induced by cerulein plus LPS (Fig. 6K). Taken together, AHCYL1 deficiency in pancreatic acinar cells induced inflammation through SSAT up-regulation in macrophages.

### Deubiquitinase OTUD6B dissociation resulted in AHCYL1 down-regulation in AP

**Fig. 7:**
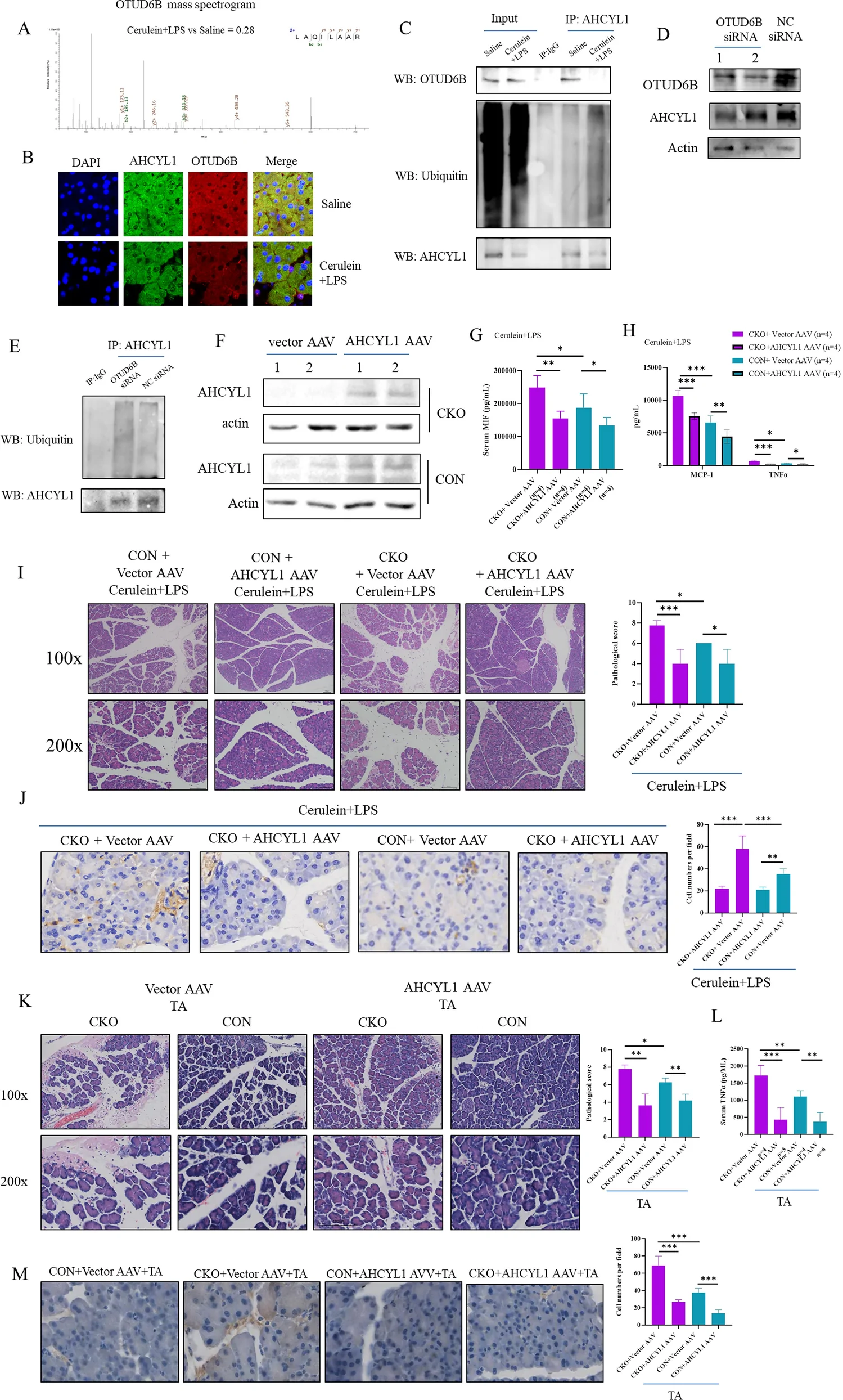

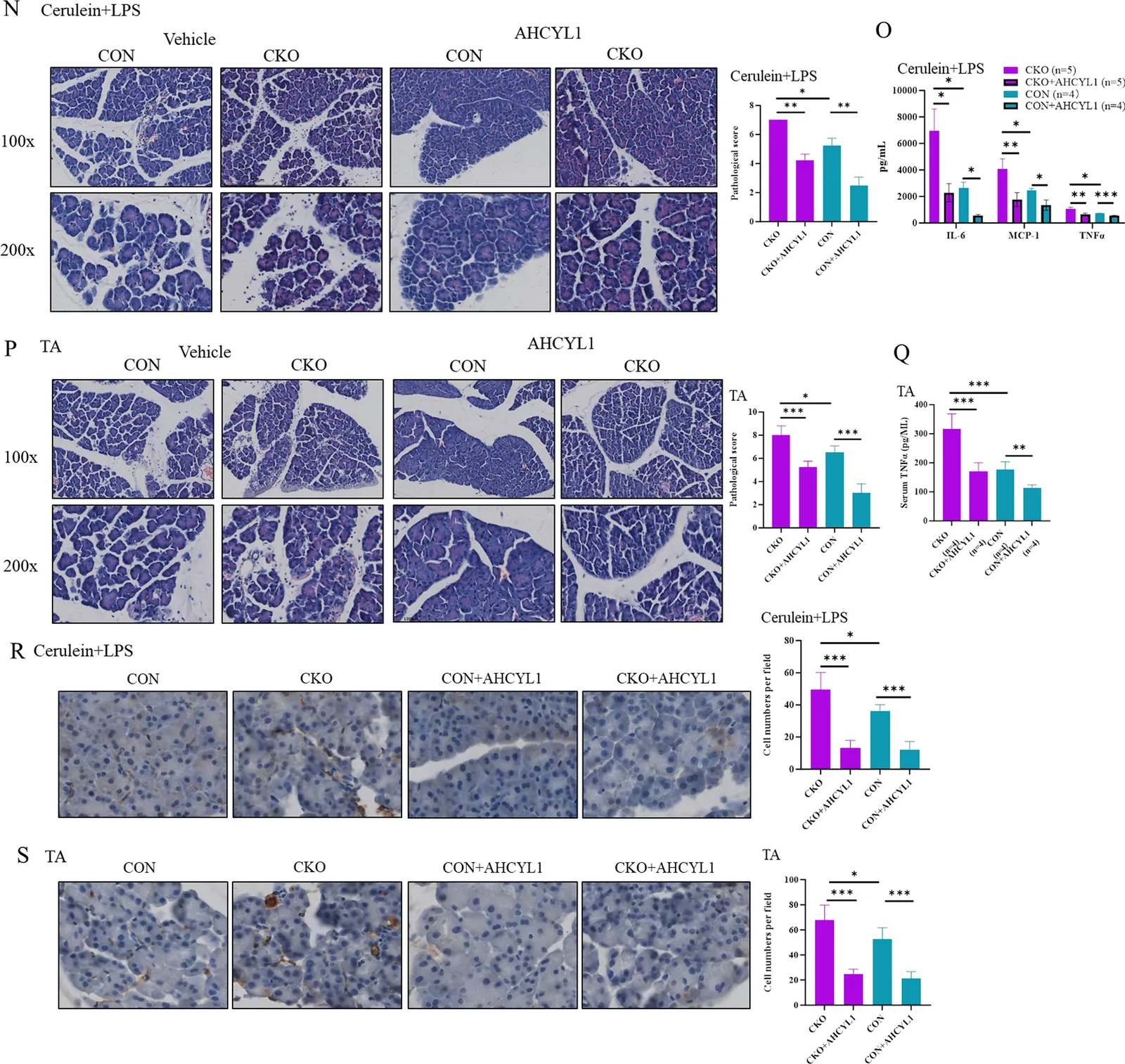
Deubiquitinase OTUD6B protected AHCYL1, and AHCYL1 recovery alleviated AP. **A**, **B** AP was induced in C57BL/6 mice. Pancreas tissues from saline or cerulein plus LPS groups were lysed, and lysates were incubated with AHCYL1 antibodies. AHCYL1 binding proteins were enriched and sent for mass spectrum analysis (A), immunofluorescence (**B**) or western blotting (**C**). OTUD6B mass spectrogram was shown (**A**). **D**, **E** Control siRNA or OTUD6B siRNA was transfected into pancreatic acinar cells for 72 h. Cells were lysed, and lysates were used for western blotting (**D**) or immunoprecipitation assay with AHCYL1 antibody (**E**). **F**–**M** Control and CKO mice were injected with vector AAV or AHCYL1 AAV for 4 weeks. AP was induced in these mice. **N**–**S** Mice were injected with recombinant AHCYL1 after AP induction. Pancreas tissues were lysed, and lysates were analyzed by western blotting (**F**). Cytokines in serum were detected with ELISA (**G**) or flow cytometry (**H**, **L**, **O** and **Q**). Pancreas tissue sections were sent for HE staining (**I**, **K**, **N** and **P**) and immunochemistry (**J**, **M**, **R** and **S**). The number of CD68-positive macrophages in each sample was evaluated as the mean value from five fields using Image J. Graphs are presented as mean ± SD, ^*^p < 0.05, ^**^p < 0.01, ^***^p < 0.001

The regulation of AHCYL1 in AP was not clear. Apart from SLC7A5 and SLC7A8, OTUD6B which is a deubiquitinase was also found to dissociate with AHCYL1 in the co-immunoprecipitation mass spectrum (Fig. 7A). The IP signaling ratio of OTUD6B was equal to 0.28 indicating that the interaction between OTUD6B and AHCYL1 decreased under cerulein plus LPS treatment (Fig. 7A). Immunofluorescence and western blot results validated the mass spectrum data (Fig. 7B and 7C). Meanwhile, cerulein plus LPS increased the ubiquitination of AHCYL1 (Fig. 7C). Moreover, the protein expression of AHCYL1 decreased when OTUD6B was knocked down (Fig. 7D). The ubiquitination of AHCYL1 also increased in the case of OTUD6B knockdown (Fig. 7E). These results suggested that decreased OTUD6B binding to AHCYL1 resulted in the latter’s down-regulation in AP.

### AHCYL1 complement ameliorated AP

AHCYL1 deficiency resulted in a stronger inflammation reaction in AP. Thus the effect of AHCYL1 recovery in AP was investigated. AHCYL1 recovery in the pancreas of control and AHCYL1 CKO mice was managed by AHCYL1-encoding retrovirus (pancreas-specific) infection. AHCYL1 was truly recovered (Fig. 7F). It was found that AHCYL1 recovery inhibited MIF and cytokines in both control and AHCYL1 CKO mice with AP (Fig. 7G, 7H and 7L). HE staining showed that injury in the pancreas was inhibited in both types of mice injected with AHCYL1 virus (Fig. 7I and 7K). Consistently, the number of CD68 + macrophages infiltrated in the pancreas also decreased under AHCYL1 virus treatment (Fig. 7J and 7M).

In order to investigate the roles of AHCYL1 in AP, recombinant AHCYL1 was used directly. Consistent results were observed (Fig. 7N–S). These results indicated that AHCYL1 ameliorated AP.

## Discussion

In this research it was found that AHCYL1 was decreased in AP. AHCYL1 specifically knocked out in pancreatic acinar cells caused macrophage infiltration to increase. The dissociation between AHCYL1 and SLC7A5 or SLC7A8 resulted in amino acid uptake inhibition followed by mTOR inactivation. mTOR inhibition in pancreatic acinar cells induced PGK1, and then MIF expression increased. MIF release from acinar cells caused macrophage infiltration and cytokine secretion from macrophages. Moreover, the effect of MIF on the macrophages was through SSAT up-regulation. Finally, AHCYL1 downregulation was due to its dissociation from deubiquitinase OTUD6B followed by ubiquitin-mediated degradation. AHCYL1 recovery reduced the inflammatory reaction in AP.

Amino acid transportation was not fully understood in acute pancreatitis. It has been recently reported that amino acids including glutamate and glycine were decreased in acinar cells during AP [32]. Consistently these amino acids’ uptake was inhibited in pancreatic acinar cells treated with cerulein plus LPS. Meanwhile, during the AP SLC7A5 and SLC7A8 were impaired [14]. While our study found that the interaction between AHCYL1 and SLC7A5 or SLC7A8 could regulate amino acid uptake. Taken together, it could be hypothesized that both AHCYL1 down-regulation and SLC7A5 or SLC7A8 impairment promote the development of AP. Besides, SLC7A5 and SLC7A8 were found to be located mainly in the cell membrane and partially in the cytoplasm in acinar cells [14]. However, these two transporters were found to be mainly in the cytoplasm in acinar cells. In other types of cells, SLC7A8 could exist in the cytoplasm [33, 34]. It was reported that amino acid transporters’ localization changed with different stimuli [35]. Therefore, the presence of SLC7A5 or SLC7A8 in acinar cell cytoplasm was rational. The events related to the cytoplasmic localization of SLC7A5 or SLC7A8 in acinar cells need to be investigated further.

EIF2α phosphorylation and ATF4 increment indicated protein synthesis inhibition in AP. This phenomenon is further evidence of amino acid uptake inhibition in AP. Meanwhile, it was consistent with the previous report [36]. However, we found amylase in acinar cells with AP to be increased. It might be due to the degradation of amylase inhibition.

In our research, amino acid uptake inhibition reminded us to detected mTOR. In the study, uptake of both essential and non-essential amino acids was inhibited. It’s common that essential amino acid deprivation resulted in mTOR inactivation [37]. Acinar cells possessed strong ability of protein synthesis [38]. Therefore, exogenous non-essential amino acids might be important for protein synthesis in acinar cells. Then non-essential amino acid deprivation might also cause mTOR inactivation in AP. Actually the roles of mTOR were not understood fully. Some papers indicated that mTOR was inhibited in AP [39–41]. While some papers found that mTOR was activated in AP [17, 42]. In a recent report, time-course study of pancreatic mTOR was performed [18]. It was found that mTOR phosphorylation decreased short time after AP induction. However, long time cerulein treatment induced mTOR phosphorylation increment. It was reported that both autophagy induction increment and autophagy flux blockage occurred in AP [43]. mTOR phosphorylation decrement representing inactivation can result in autophagy induction up-regulation [44]. Increased mTOR phosphorylation usually results in cell proliferation [45]. In the later stage of AP, pancreas recovery was started and mTOR might worked during this stage. Our results found that mTOR phosphorylation inhibition in AP which might be the event present in the early stage of AP.

In the PPI network, the direct association of mTOR and PGK1 was found. An mTOR activator could result in an increase in PGK1 protein. PGK1 was the key enzyme in glycolysis and contributed to ATP production by transferring a phosphate group to ATP [46]. Whether the roles of PGK1 in AP were related to ATP production was not understood. Besides, PGK1 also worked as a protein kinase and regulated various biological activities including autophagy [47]. Under glutamate deprivation, mTOR was inhibited followed by PGK1 acetylation. Acetylated PGK1 resulted in Beclin1 phosphorylation and autophagy up-regulation in tumor cells [48, 49]. In our research, mTOR was inhibited and PGK1 was induced by mTOR inhibition. Therefore, mTOR/PGK1 might work as the upstream regulator for autophagy in AP. It has been reported that post-translational modification of PGK1 affects its degradation [50]. Therefore mTOR might affect the PGK1 phosphorylation and then the stability of the latter. Moreover, PGK1 could affect mTOR [51]. This feedback regulation might also be present in AP which needs to be investigated further.

It was known that the chemotactic function of MIF results in the recruitment of monocytes/macrophages to the inflammatory site [28]. It is necessary to mention here that monocytes from the blood contribute to the severity of AP [52]. We found MIF could induce macrophage migration. Taken together, it was thought that MIF could promote blood monocyte recruitment to the pancreas and macrophage migration to the injured pancreatic cells in AP.

In this research, PGK1 induced MIF expression. While, it was reported that MIF positively regulated the mRNA expression of PGK1 [53]. The mechanism underlying PGK1 induced MIF was probably related to transcription regulation since NFkb was inhibited by PGK1 inhibitor. This hypothesis was consistent with a report that PGK1 inhibitor inhibited the transcription of pro-inflammatory cytokine, IL-6 in LPS-stimulated macrophages [54]. It was recently found that PGK1 protein expression was increased under the treatment of cerulein and PGK1 inhibitor could alleviate AP including attenuating inflammatory [55]. Of note, PGK1 inhibition reduced macrophages infiltration. Therefore, our results were consistent with the previous report.

In early research, it was found that SSAT activity was increased in AP and SSAT activation resulting in N^1^-acetylspermine increment caused acute pancreatitis including pancreatic inflammation [56]. Consistently, SSAT was upregulated in macrophages further induced by MIF secreted from AHCYL1 knockout acinar cells. How MIF induced SSAT was not really understood here. N^1^-acetylspermine belonged to polyamines. A previous report showed that other polyamines including spermine and spermidine, precursors for N^1^-acetylspermine, decreased in serum from AP mice [57]. However, in this study other polyamine metabolites did not change in AHCYL1 CKO mice with AP compared with the control. There might be other enzymes working here to prevent the decrement of spermine or spermidine. In this study, N^1^-acetylspermine was chosen, but other serum metabolites might also work in acute pancreatitis.

The roles of OTUD6B in acute pancreatitis were not clear. Our study indicated that OTUD6B might ameliorate acute pancreatitis by stabilizing AHCYL1. AHCYL1 was known to regulate homocysteine metabolism [58], ion transportation [5], but it was found to regulate amino acid transportation here. Mist1 knockout diminished the decrement in the OTUD6B mRNA expression in the pancreas under cerulein treatment according to the RNA sequencing dataset GSE3644. Therefore, Mist1 might be the upstream regulator of OTUD6B in our article.

## Conclusions

Our study showed that deubiquitinase OTUD6B disassociated with AHCYL1 resulting in an AHCYL1 decrement followed by amino acid uptake inhibition and mTOR inhibition in AP. Inactivated mTOR induced PGK1 and MIF expression increased. MIF secretion from acinar cells induced SSAT in macrophages and macrophage infiltration in the pancreas increased. AHCYL1 deficiency in acinar cells strengthens all of the signaling.

## Supplementary Information

Below is the link to the electronic supplementary material.

Supplementary file1 (DOCX 435 KB)

## Abbreviations

AP: Acute pancreatitis
AHCYL1: S-adenosylhomocysteine hydrolase-like protein 1
IRBIT: IP_3_R binding protein released with inositol 1,4,5-trisphosphate
MIF: Macrophage migration inhibitory factor
SSAT: Spermidine/spermine N^1^-acetyltransferase
H&E: Hematoxylin and eosin
SD: Standard deviation
LPS: Lipopolysaccharide
