# Supplementary material for: S-adenosylhomocysteine hydrolase-like protein 1 deficiency resulted in stronger inflammation reaction in acute pancreatitis
Source: J Gastroenterol. 2026 May 9;61(10):1483–501. doi: 10.1007/s00535-026-02437-x (PMC13616016; doi:10.1007/s00535-026-02437-x)
Supplement: Supplementary file 1 [file 535_2026_2437_MOESM1_ESM.docx]

S-adenosylhomocysteine hydrolase-like protein 1 deficiency resulted in stronger inflammation reaction in acute pancreatitis

supplementary figures

**Figure S1. Genotype identification in mice.**

**Figure S2. AHCYL1 deficiency didn’t affect digestive enzymes and cytokines under saline treatment.** AP was induced in control or CKO mice, serum from saline treated mice were sent for biochemical analysis (A) and cytokines measurement (C). Serum from cerulein plus LPS treated mice were sent for biochemical analysis (B).

**Figure S3. AHCYL1 deficiency didn’t affect T cells and neutrophils infiltrations in pancreas.** AP was induced in control or CKO mice, pancreas were sent for immuochemistry. CD3 positive T cells (A) or MPO positive neutrophils (B) number in each sample was evaluated as mean value from five fields using Image J. Graph presented as mean ± SD, ns non significance, ^*^ *P* < 0.05, ^**^ *P* < 0.01, ^***^ *P* < 0.001.

**Figure S4. Amylase increased further AHCYL1 deficiency acinar cells with AP.** Primary acinar cells from control or AHCYL1 CKO mice were treated with or without cerulein plus LPS. Cells were lysed and analyzed by western blotting.

**Figure S5. MIF expression pattern.** Pancreas from C57BL/6 mice were digested and sent for single cell RNA sequencing.

**Figure S6. Metabolites secreted from AHCYL1 deficiency acinar cells didn’t affect SSAT and PAOX.** Microphages were treated with cerulein plus LPS in the presence or absence of indicated metabolites for 6h. Cells were lysed and analyzed by western blotting.
